# Nanoparticles as Adjuvants and Nanodelivery Systems for mRNA-Based Vaccines

**DOI:** 10.3390/pharmaceutics13010045

**Published:** 2020-12-30

**Authors:** Iman M. Alfagih, Basmah Aldosari, Bushra AlQuadeib, Alanood Almurshedi, Mariyam M. Alfagih

**Affiliations:** 1 Department of Pharmaceutics, College of Pharmacy, King Saud University, Riyadh 11671, Saudi Arabia; baldosari@ksu.edu.sa (B.A.); bquadeib@ksu.edu.sa (B.A.); marshady@ksu.edu.sa (A.A.); 2 Department of Pharmaceutical Sciences, College of Pharmacy, Aalfaisal University, Riyadh 11533, Saudi Arabia; malfagih@alfaisal.edu

**Keywords:** mRNA, adjuvant, vaccine, nanoparticles, nanodelivery systems, lipids, polymers

## Abstract

Messenger RNA (mRNA)-based vaccines have shown promise against infectious diseases and several types of cancer in the last two decades. Their promise can be attributed to their safety profiles, high potency, and ability to be rapidly and affordably manufactured. Now, many RNA-based vaccines are being evaluated in clinical trials as prophylactic and therapeutic vaccines. However, until recently, their development has been limited by their instability and inefficient in vivo transfection. The nanodelivery system plays a dual function in RNA-based vaccination by acting as a carrier system and as an adjuvant. That is due to its similarity to microorganisms structurally and size-wise; the nanodelivery system can augment the response by the immune system via simulating the natural infection process. Nanodelivery systems allow non-invasive mucosal administration, targeted immune cell delivery, and controlled delivery, reducing the need for multiple administrations. They also allow co-encapsulating with immunostimulators to improve the overall adjuvant capacity. The aim of this review is to discuss the recent developments and applications of biodegradable nanodelivery systems that improve RNA-based vaccine delivery and enhance the immunological response against targeted diseases.

## 1. Introduction

Messenger RNA (mRNA)-based vaccines have shown promise as techniques for the development of vaccines against infectious diseases and several types of cancer since the 2000s. They have been evaluated in multiple studies on the prophylaxis and treatment of infectious diseases, several types of cancer, and autoimmune diseases. Clinical studies have demonstrated their potential in terms of safety and immune responses. As prophylactic vaccines in infectious diseases, clinical trials are still in their early stages, mostly phase I clinical studies, with most trials testing mRNA-based vaccines for coronavirus disease 2019 (COVID-19) and rabies. Other studied conditions include Zika virus and H10N8 and H7N9 influenza viruses. As therapeutic vaccines for cancer immunotherapy, many mRNA-based vaccines have proceeded to phase II clinical trials, including those for melanoma, non-small-cell lung carcinoma, and prostate cancer. Examples of clinical trials and their results are summarized in Table 1.

**Figure 1:**
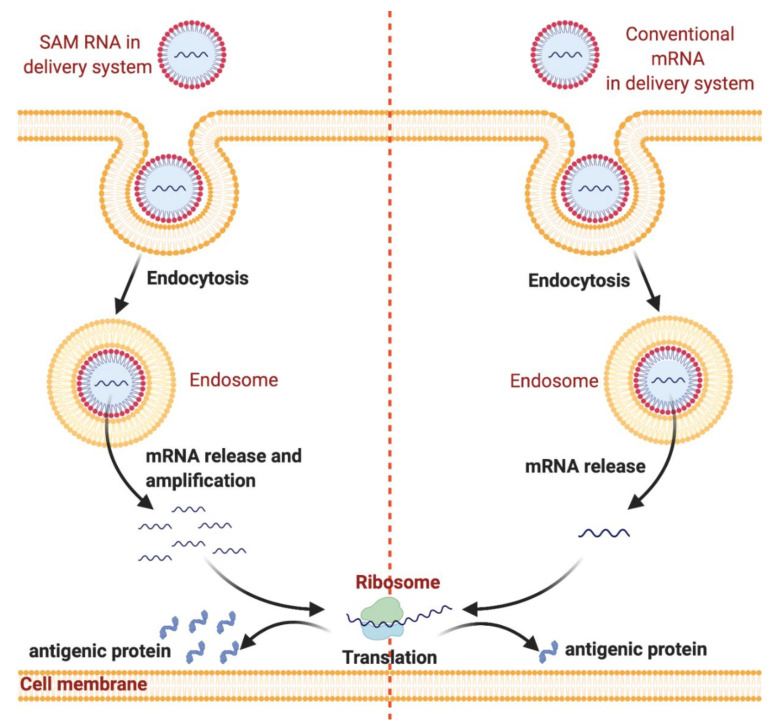
Nanodelivery systems protect messenger RNA (mRNA) from degradation and enhance endocytosis and endosomal escape. mRNA can be loaded into nanodelivery systems for protection from enzymatic degradation. After administration, mRNA is internalized by dendritic cells through endocytosis followed by endosomal escape. Subsequently, mRNA is released into the cytosol, which is followed by mRNA translation into antigenic protein by ribosomes. The figure was created using Biorender.com.

mRNA-based vaccines work by using the host cell’s translation mechanism to produce the relevant antigen and trigger the adaptive immune response. After entering the cytosol, the cell treats it as an endogenous mRNA and translation starts instantly (Figure 1) [1].

**Figure 2:**
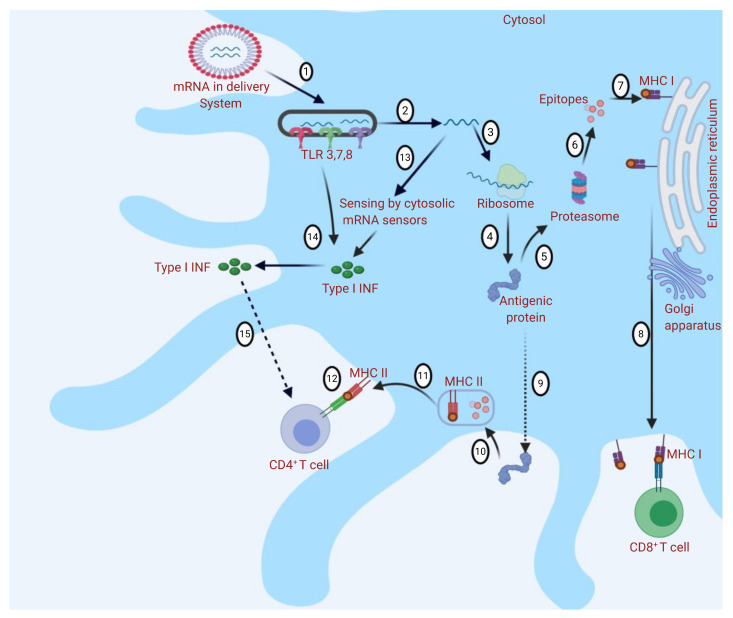
Different immune responses to messenger RNA (mRNA)-based vaccine nanodelivery systems: The mRNA delivery system is engulfed into the cell by endocytosis, and Toll-like receptors (TLRs) are activated (1). mRNA is released into the cytosol (2). mRNA binds to ribosome and translation occurs (3 and 4). Antigenic protein provokes the immune response through major histocompatibility complex (MHC I or MHC II) presentation (5 and 9). (5) In MHC I presentation, peptides are produced by proteolysis (6), and CD8+ T cells are stimulated (7 and 8). In MHC II presentation (9), peptides are produced by endosomal proteolysis (10 and 11), and CD4+ T cells are stimulated (12). Cytosolic sensing of intracellular mRNA leads to activation of retinoic acid-inducible gene (RIG)-I-like receptors (RLRs) and nucleotide-binding oligomerization domain (NOD)-like receptors (NLRs). Activation of TLRs, RLRs, and NLRs induces the production of type I interferons (IFNs) (13 and 14). Type I IFNs may have a positive or negative effect on the activation of T cells (as they could determine the differentiation of antigen-primed CD8^+^ T cells into cytotoxic effectors but may also cause T-cell exhaustion) (15). The figure was created using Biorender.com.

The immune system can also be triggered when extracellular mRNA is recognized by pattern recognition receptors (PRRs): endosomal membrane-located Toll-like receptors (TLRs 3, 7, and 8) and cytosolic nucleic acid sensors such as retinoic acid-inducible gene (RIG)-I-like receptors (RLRs) (Figure 2). When activated, PRRs provoke type I interferon (IFN) responses, which may be strong and negatively affect antigen expression and may cause T-cell exhaustion. However, the formulation of the vaccine impacts the types of sensors activated. During the in vitro production, mRNA capping is necessary to be recognized as an endogenous molecule. This could be achieved using cap analogues or through enzymatic capping. Double-stranded RNAs (dsRNA) resulting from the transcription reaction should be removed to reduce type I IFN production and enhance antigen expression through high-performance liquid chromatography (HPLC) or cellulose purification [10]. mRNA vaccines can be divided into two types: conventional mRNA-based vaccines and self-amplifying mRNA (SAM RNA) vaccines. Conventional mRNA-based vaccines harbor only the antigen gene of interest and cannot amplify itself, whereas the SAM RNA vaccines each contain an engineered RNA virus genome that comprises the virus’s non-structural protein genes, which are essential in the RNA replication machinery, and the antigen gene of interest. After its introduction into the host cell, a SAM vaccine is able to amplify, resulting in replicons that are able to produce high amounts of the antigen gene. Replicons are unable to produce infectious virions as they lack the structural protein genes, and thus, cannot spread to the neighboring cells [11]. SAM RNA vaccines are designed to enhance the extent of protein expression and the induced immune response. The use of mRNA-based vaccines has multiple advantages over conventional whole-organism, subunit, and DNA-based vaccines, including safety profile, ability to be rapidly developed, low-cost manufacturing potential, and high potency. mRNA-based vaccines are considered to be relatively safe as there is no associated risk of infection as experienced with live attenuated vaccines [12]. Additionally, compared to DNA-based vaccines, there is no need for the mRNA to enter the nucleus; thus, there is no risk of genomic integration and unpredictable long-term expression is avoided, allowing control of the treatment duration and side effects [13]. Moreover, mRNA production is cell-free, using in vitro transcription (IVT) methods that highly decrease bacterial contamination and permit rapid scale-up over a short period and low-cost manufacturing due to the high yields [1]. A new purification method using cellulose have been developed recently which helps in further reducing the manufacturing costs. In comparison to HPLC, this method does not require high-cost equipment, fast, with similar antigen expression and more mRNA recovery rate in comparison to mRNA--based vaccines purified by HPLC [14]. Thus, mRNA-based vaccines are good candidates for responding to the COVID-19 pandemic. The design and manufacturing of mRNA-based vaccines on the clinical scale is possible within weeks when the viral antigen sequence becomes available. For instance, only 42 days were required for Moderna’s mRNA-1273 (Cambridge, MA, USA) to enter phase I clinical testing after the sequencing of the full SARS-CoV-2 genome (ClinicalTrials.gov identifier NCT04283461). Recently, mRNA-1273 has entered into phase III clinical studies (ClinicalTrials.gov identifier NCT04470427).

Until recently, mRNA vaccine applications have been restricted by instability and inefficient in vivo delivery of mRNA molecules. Their delivery to the cytosol is hindered by the rapid degradation of naked mRNA by ribonuclease and the negatively charged and high-molecular-weight (10^5^–10^6^ Da) mRNA molecule, preventing passive diffusion across the cell membrane [15,16]. Although local intranodal injection of RNA leads to the effective activation of a specific immune response [17,18,19], delivery systems are crucial for successful in vivo delivery of mRNA to the site of action and for large-scale preventive vaccinations.

**Figure 3:**
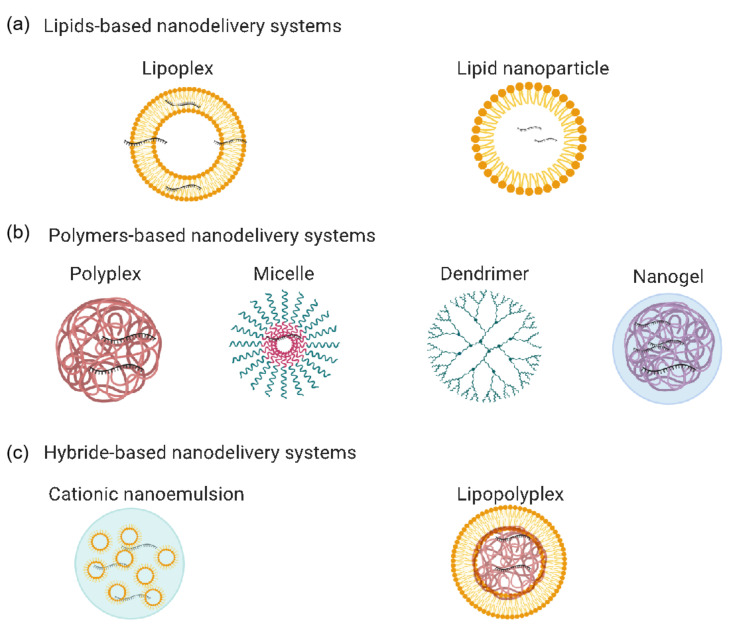
mRNA-based vaccine nanodelivery systems: (**a**) lipid-based nanodelivery systems, including lipoplexes and lipid nanoparticles; (**b**) polymer-based nanodelivery systems, including polyplexes, micelles, dendrimers, and nanogels; (**c**) hybrid-based nanodelivery systems, including cationic nanoemulsions and lipopolyplexes. The figure was generated using Biorender.com.

Ideally, the in vivo delivery system should provide protection against degradation of the mRNA molecule by extracellular RNAses, facilitate cellular uptake, and target antigen-presenting cells (APCs). Multiple methods have been studied to enhance mRNA delivery. These include physical methods, viral-based vectors, and non-viral vectors. Physical methods include using a gene gun and electroporation; both methods have been tested and shown to improve delivery to cells [20,21]. However, physical methods often destroy the cells, making them unsuitable for mRNA delivery [22,23,24]. Viral-based vectors have also been studied as mRNA delivery systems, wherein the genes of adeno-associated viruses are replaced completely or partially with the gene of interest. A drawback of this method is the risk of genomic integration [25,26,27]. Non-viral victors include nanodelivery systems (Figure 3, Table 2): lipid-based, polymer-based, and lipid–polymer hybrid nanoparticles [28,29,30,31,32,33,34]. These are currently the most favored methods for delivering mRNA, as they are considered safe, stable, and low-cost, and provide highly efficient transfection. They provide the advantage of acting as delivery systems and as adjuvants. Due to their resemblance to pathogens in size and structure, nanoparticles can induce immunogenicity by mimicking the natural infection activity. Nanodelivery systems also allow non-invasive mucosal administration, targeted immune cell delivery, and controlled delivery, reducing the need for multiple administrations. Moreover, they allow co-encapsulating with immunostimulators to improve the overall adjuvant capacity [27]. Furthermore, it was reported that when nanoparticles (of 10–100 nm size) injected into tissue (i.e., through intramuscular, intradermal, subcutaneous or intraperitoneal) they are readily carried across the lymphatic endothelium by interstitial fluid, but they are too big to diffuse into the blood circulatory system. These nanoparticles are found to have enhanced lymph node transport efficiencies which is very important for vaccine developments [34]. Because improved lymphatic transport permits direct access to dendritic cells in lymphoid node and consequently facilitates the antigen uptake and presentation to B cells enhancing the antibody-mediated immunity [35], or to T cells, allowing cell-mediated immunity [36,37]. More details on the importance of the nanodelivery systems in the vaccines development have been described in recent reviews [38,39].

The aim of the rest of this review is to discuss the recent applications of biodegradable nanoparticles that improve RNA-based vaccine delivery and enhance the immunological response against targeted diseases.

## 2. mRNA-Based Vaccines’ Nanodelivery Systems

### 2.1. Lipid-Based Nanodelivery Systems

Currently, lipid nanoparticles and lipoplexes are among the most commonly used lipid-based nanodelivery systems for in vivo delivery of mRNA molecules [29]. The general chemical structure of a cationic lipid consists of a hydrophobic chain attached to a head group. The chain is a hydrophobic chain that is symmetric or di-symmetric, modified or unmodified, saturated or unsaturated, and linear or branched. The cationic head group holds a linker molecule that can be cleavable to enhance biodegradation. The head group usually holds at least one group, for instance, amine, which ionizes and changes its negative charge to a positive charge at physiological pH. Accordingly, cationic lipids can be complexed with anionic molecules via electrostatic interactions [47].

#### 2.1.1. Lipid-Nanoparticle-Based Nanodelivery Systems

Lipid nanoparticles are often formulated using cationic or ionizable cationic lipids, cholesterol, phospholipids, and lipid-anchored polyethylene glycol. Cationic lipids allow spontaneous association of negatively charged mRNA, via a combination of attractive electrostatic interactions with mRNA and hydrophobic interactions, which forms a core onto which the other lipids associate when their solubility limit is reached. This, in turn, and enhance mRNA endosomal release to the cytoplasm. Cholesterol is incorporated as a stabilizing agent and phospholipids are incorporated to support the lipid bilayer structure [73]. Lipid-anchored polyethylene glycol (lipid-PEG) is incorporated to increase the half-lives of formulations, to reduce nonspecific interactions with plasma proteins and to provide steric stabilization of the formulation before use [74,75]. The right amount of lipid-PEG coating on the lipid nanoparticles is crucial. The addition of higher amount of lipid-PEG usually increases the blood circulation time of lipid nanoparticles and consequently reduces cellular uptake and interaction with endosomal membrane [76]. However, it was reported that anti-PEG antibody response following repeated intravenous administration of PEGylated lipid nanoparticles dramatically accelerated blood clearance of the lipid nanoparticles and lead to acute hypersensitivity [77]. Furthermore, many reports demonstrated that lipid-PEG coating clearly enhances lymphatic drainage [78,79]. However, enhanced lymphatic drainage does not necessary lead to a more potent immune response. The noticed improvement in lymphatic drainage could be attributed to a higher shielding of the cationic charges of lipid nanoparticles against unspecific interactions with proteins [80]. These results are very important for immunotherapy applications, where multiple doses are necessary for long-term protection.

Many studies have reported efficient in vivo small interfering RNA (siRNA) delivery by lipid nanoparticles [81]. In 2018, the siRNA–lipid nanoparticles delivery system was approved as part of the product Patisiran (Onpattro, Alnylam Pharmaceuticals, Cambridge, MA, USA) for patients with hereditary transthyretin-mediated amyloidosis to inhibit hepatocyte expression of transthyretin [82]. However, it has only recently been demonstrated that lipid nanoparticles are efficient in vivo delivery systems for SAM RNA and mRNA [15]. The delivery of mRNA via lipid nanoparticles is proposed to occur through endocytosis followed by electrostatic attachment and fusion with the cell membrane through inverted non-bilayer lipid phases [15]. In addition to their roles in the protection of mRNA, lipid nanoparticles enhance cellular uptake and endosomal escape, enabling cytoplasmic delivery [15]. Another important advantage of lipid nanoparticles for vaccination is the adjuvant activity that evokes the immune system and induces inflammation [83]. The endosomal escape is governed by the properties of the ionizable lipid such as structure of the hydrophobic domain, the ionizable lipid’s pKa and the molar ratio between ionizable lipids and mRNA nucleotides. This is discussed in more detail later. However, only a small percentage (up to 15%) of the total mRNA loaded into the internalized lipid nanoparticles is reported to actually reach the cytoplasm through this pathway depending on the fusogenicity of the ionizable lipid used [84,85]. Maugeri et al. reported that the mRNA which was not released intracellularly was instead packed into endosomal intra-luminal vesicles and secreted outside the cells loaded into extracellular vesicles. Human erythropoietin (hEPO) mRNA–lipid nanoparticles were transfected in human epithelial HTB-177 cells. Exosomes carrying hEPO mRNA and ionizable lipids (at a molar ratio of 1:1) were released by the transfected cells. However, when the hEPO mRNA–lipid nanoparticles pre-incubated with exosomes, lipid nanoparticles did not fuse with exosomes outside the cell, but instead processed in the endosomal pathway and were released in endo-exosomes which indicates a connection between the endocytosis of the lipid nanoparticles and the exocytosis of mRNA. Furthermore, in vivo studies demonstrated that the mRNA-loaded exosomes administered intravenously were able to deliver active mRNA leading to hEPO protein production in different organs (mainly in the liver) although lower than the level of protein produced by lipid nanoparticles. Also, the mRNA-loaded exosomes induced significantly lower inflammatory cytokine production than the lipid nanoparticles due to the lower amount of ionizable lipids present [86].

Ionizable lipids applied for mRNA delivery are neutral at physiological pH, which is suitable for tolerability and the safety profile after vaccination. However, they are positively charged at low pH, which allows mRNA complexation under acidic conditions and enhances their electrostatic interaction and fusion with the negatively charged endosomal membrane [15].

Lutz et al. developed lipid nanoparticles for delivering mRNA encoding rabies or influenza antigens. The results revealed that the formulations are well tolerated and highly immunogenic in non-human primates. The immunization of non-human primates via intramuscular injection with these formulations evoked protective antibody titers. The results demonstrated that the optimized formulations are similar to vaccinations with licensed formulations based on inactivated virus in terms of humoral and cellular immune responses. The immune responses were boosted and stayed active during a period of up to 1 year. Moreover, the optimized formulations exhibit a favorable safety profile [40]. 

Recently, Hassett et al. assessed a group of proprietary biodegradable ionizable lipids in addition to distearoylphosphatidylcholine (DSPC), cholesterol, and polyethylene glycol (PEG) lipid to develop lipid nanoparticles encapsulating mRNA encoding firefly luciferase and the H10N8 influenza hemagglutinin antigen. These lipid nanoparticles were evaluated for both expression and immunogenicity in a murine model after intramuscular administration. A subset of five mRNA–lipid nanoparticles was chosen and further assessed for expression, immunogenicity, and tolerability in rats and monkeys. A selected formulation was identified that produced a robust immune response with enhanced safety. For vaccines, the increase in innate immune stimulation triggered by lipid nanoparticles was not associated with an increase in immunogenicity, demonstrating that mRNA vaccine safety can be enhanced without affecting efficiency [41].

More recently, Zhang et al. applied ionizable lipid, 1,2-distearoyl-sn-glycero-3-phosphocholine (DSPC), cholesterol, and PEG lipid (with molar ratios of 50:10:38.5:1.5) to formulate a lipid nanoparticle-encapsulated mRNA vaccine encoding the receptor-binding domain of SARS-CoV-2 (ARCoV). This formulation was administered to mice and non-human primates via the intramuscular route, leading to robust neutralizing antibodies against SARS-CoV-2 as well as a Th1-biased cellular response. Additionally, two doses of ARCoV immunization in mice afforded full protection in a SARS-CoV-2 mouse-adapted strain challenge model. ARCoV is available as a liquid preparation and it is stable at 4 °C and 25 °C for at least 7 days. Furthermore, 1000 μg of ARCoV did not cause obvious adverse effects, highlighting the safety of this formulation. ARCoV was approved for phase I clinical trials (ChiCTR2000034112) in June 2020 [42]. 

Although ionizable lipids are applied as components of mRNA–lipid nanoparticles, they may be, in some cases, notably more expensive than available cationic lipids such as 1,2-dioleoyl-3-trimethylammonium-propane (DOTAP). Less clinical data are available on the use of novel ionizable lipids, which is important from regulatory and safety perspectives [45]. In contrast, lipid nanoparticles formulated using cationic lipids have been extensively studied for delivery of subunit antigens [87], DNA [88], and SAM RNA [89], and have an acceptable safety profile. Accordingly, lipid nanoparticle formulations based on well-established lipids could enhance and hasten the pharmaceutical development of mRNA and SAM RNA vaccines [45].

Lou et al. investigated a group of conventional cationic lipids to develop a SAM RNA vaccine delivery system. They compared the cationic lipid nanoparticles formulated with the benchmark ionizable lipid nanoparticles described by Geall et al. [90]. SAM RNA encoding the rabies virus glycoprotein (RVG) was encapsulated in cationic lipid nanoparticles with 70–99% encapsulation efficiency. They found that the higher transfection efficiency of dimethyldioctadecylammonium (DDA)- and DOTAP-containing lipid nanoparticles in comparison with other cationic lipids was directly related to the ability of DDA and DOTAP to pack and stabilize SAM RNA. DDA- and DOTAP-containing lipid nanoparticles were superior to benchmark ionizable lipid nanoparticles. This could be due to higher cellular association of DOTAP and DDA–lipid nanoparticles in comparison with ionizable lipid nanoparticles. In vitro toxicity studies demonstrated no cytotoxicity in the range of SAM RNA lipid nanoparticles concentrations tested. In vivo studies demonstrated that DDA–cationic lipid nanoparticles remained longer at the injection site compared to DOTAP–lipid nanoparticles and ionizable lipid nanoparticles after intramuscular injection in mice. Both the cationic lipid nanoparticles and the ionizable lipid nanoparticles evoked strong humoral and cellular-mediated immune responses in mice that were not significantly different at a 1.5 μg SAM RNA dose [45].

Due to promising preclinical results, many lipid-nanoparticle-based mRNA vaccines have entered clinical studies to evaluate their effectiveness (Table 1). Bahl et al. revealed that a single intradermal injection with lipid-nanoparticles-encapsulated mRNA encoding the nucleoside-modified hemagglutinin (HA) gene of H10N8 or H7N9 at 10 μg led to the production of HA antibodies in mice for more than a year. This nanodelivery system was evaluated in human volunteers (phase I clinical trial; NCT03076385). The data demonstrated that after two intramuscular immunizations (100 μg) separated by a 3-week interval, the vaccine was well tolerated and able to produce robust humoral immune responses [91]. Similarly, Feldman et al. demonstrated that intramuscular injections with lipid nanoparticle-encapsulated mRNA encoding the nucleoside-modified HA genes of H10N8 and H7N9 influenza strains respectively at two doses 3 weeks apart and at 100 and 25 μg doses were safe and able to produce robust humoral immune responses in healthy adults [3].

Targeting ligands are included in mRNA–lipid nanoparticle delivery systems to specifically recognize receptors on cells and improve cellular uptake [92]. For instance, mannose receptors are good candidate targets for vaccines to introduce genes encoding antigens because they are expressed on APCs (especially macrophages and dendritic cells) [93,94,95]. Zhuang et al. compared cationic lipid nanoparticles with mannose-conjugated cationic lipid nanoparticles regarding the efficiency of the mRNA encoding the HA protein of influenza A H1N1. The cytotoxicity studies present that when the molar of N (nitrogen on DOTAP) was less than 100 nmol/10^4^ cells, the lipid nanoparticles and mannose-conjugated cationic lipid nanoparticles had low toxicity, and the cells viability were more than 80%. The results indicated that the protein expression in the mannose-conjugated cationic lipid nanoparticle group was better than that in cationic lipid nanoparticle group both in vitro and in vivo. The immunogenicity and protective effect against the ten-fold median lethal dose (LD_50_) H1N1 influenza virus challenge in mice were evaluated two weeks after the boosting immunization. The data demonstrated that these formulations could evoke both humoral and cellular immune responses. The mRNA vaccine completely protects mice from weight loss and death [46].

#### 2.1.2. Lipoplex-Based Nanodelivery Systems

Lipoplexes are complexes formed spontaneously via electrostatic interactions between cationic lipids (especially cationic liposomes) and anionic nucleic acids. Liposomes have long been used as drug delivery systems due to their relatively easy formulation protocol, low toxicity, and biodegradability [96]. Many liposome preparations used as carriers for small molecules have been approved by the FDA [97]. Efficient and safe siRNA-liposomal formulations have been documented in human trials [98]. Different liposome preparations have been formulated to efficiently deliver genes in vivo [99], and some have been examined for mRNA vaccine delivery—demonstrating significant progress in infectious diseases [30] and cancer immunotherapy [33].

The characteristics of lipoplexes are controlled by the lipid nature and composition, which also control the strength of the electrostatic interactions between the lipid and mRNA. Furthermore, a lipid’s role cannot be inferred according to the lipid structure alone. There are no reports on real structure–activity relationships. Determining the structure–activity relationship is difficult due to the different parameters involved. The characteristics of lipoplexes that indicate their function include the size, zeta potential, homogeneity, and shape.

The tendency of lipoplexes to attach to cell surfaces and the ability to deliver and release their loads intracellularly play important roles in determining their functions. For instance, the differential efficiency of binding to dendritic cells could be explained by stronger interactions between the lipid (with amine groups) and the negatively charged mRNA, potentially producing lipoplexes with higher stability. Importantly, the lipids should enclose around the mRNA to form stable compact lipoplexes. Different lipids with different characteristics, for instance, hydrophobicity and positive charge density, would result in different configurations of packing constraints during the formation of the lipoplex, which would consequently influence the complex geometry. The addition of dioleoylphosphatidylethanolamine (DOPE) plays an important role in promoting looser interactions between the cationic lipids and mRNA, along with balancing the cationic charges and promoting endosomal membrane destabilization and intracellular release [100].

The efficient entry of protected mRNA to dendritic cells is only the first step for successful mRNA nanodelivery system vaccines and will not necessarily lead to mRNA translation. It must be followed by decompaction to facilitate the intrinsic ability of mRNA to be translated [47].

Englezou et al. screened cationic lipids with different numbers of amine groups and lipoplexes formations to enhance the transfection and translation of SAM RNA. The lipids demonstrating less efficiency for delivery were found to enhance SAM RNA translation more successfully with the absence of detectable cytotoxicity. The observed translation in vitro was confirmed by in vivo studies. The selected lipoplex formulation that demonstrated higher in vitro translation of SAM RNA in dendritic cell evoked pro-inflammatory cytokines, humoral responses, and cellular responses after subcutaneous injection with the SAM RNA-encoded influenza antigen nanoparticles in mice and in an adoptive transfer model [47].

More recently, Zhang et al. introduced a novel cationic and hydrophilic peptide with antimicrobial activity, DP7, by applying an amino-acid-based activity prediction technique. They reported that the cytotoxicity of the cholesterol-modified cation peptide DP7 (DP7-C) was very low and it has potential immunomodulatory effects [101,102]. The results demonstrated that DP7-C has dual functions as a delivery system and immune adjuvant.

As a delivery system, DP7-C can efficiently deliver antigens via caveolin- and clathrin-dependent pathways into cells. As immune adjuvant, DP7-C can activate dendritic cell maturation through evoking the TLR2–MyD88–IKK–IκB–NF-κB signaling pathway and enhancing the immune response to the neoantigen [103]. In another study, DP7-C could not deliver mRNA into cells. This was due to the inability of DP7-C to transfect the mRNA transcript into cells because the mRNA sequence being longer than the DP7-C loading capacity. Instead, Zhang et al. applied the thin-film dispersion method to modify liposomes with DP7-C. The resultant DP7-C-modified liposomes were used to deliver mRNA encoding neoantigens for individualized tumor immunotherapy. The data showed that DP7-C-modified liposomes increased the efficiency of introducing mRNA into dendritic cells in vitro and in vivo. DP7-C-modified liposomes (as immunoadjuvant) more efficiently promoted dendritic cell maturation, CD103+ dendritic cell (contributing to antigen presentation) production, and pro-inflammatory cytokine secretion than unmodified liposomes both in vitro and in vivo. Animal studies demonstrated that DP7-C-modified liposomes complexed with LL2-neoantigen-encoding mRNA significantly reduced the growth of LL2 in situ and the growth of subcutaneous tumors. Also, these formulations showed improved antigen-specific lymphocyte reactions in comparison with the liposomes–LL2 neoantigen-encoding mRNA complex formulations [48].

In general, the preparation of lipid-based nanoparticles involves the use of complicated lipid ingredients, a group of delicate and expensive instruments, and some specific skills. A simple technique that permits the simple and easy formulation of an mRNA nanodelivery system that can transfect mRNA efficiently and express protein after administration would be in high demand for research and development of vaccines. Arya et al. described a simple and effective nanodelivery system for local administration of mRNA. The authors used InstantFECT, a cationic liposome-based transfection reagent, to prepare mRNA nanocomplexes. The results demonstrated high levels of expression of reporter proteins after intratumoral and intramuscular injections that lasted for at least 96 h. Modified mRNAs encoding *Staphylococcus aureus* adenosine synthase A (AdsA) and a model tumor-associated antigen ovalbumin nanocomplex were administered by a subcutaneous and intramuscular route, which efficiently elicited strong T-cell responses. Moreover, protective and therapeutic therapy with the ovalbumin mRNA nanocomplex significantly inhibited B16-ovalbumin tumor progression, accompanied by a 100% survival rate over an extended period (at least 3 months). There was no sign of obvious toxicity after mRNA–liposome nanocomplex’s administration either in vitro or in vivo [49].

### 2.2. Polymer-Based Nanodelivery Systems

Diethylaminoethyl (DEAE) dextran is a polycationic derivative of the carbohydrate polymer dextran. It was the first cationic polymer to be examined as a delivery system for mRNA [104]. Later, it was demonstrated that mRNA transfection through lipid-based delivery systems is 100 to 1000 times more efficient than DEAE–dextran-based delivery systems [105]. This finding slowed the advancement of polymer-based delivery systems and promoted the progress of lipid-based delivery systems for mRNA and other nucleic acids. However, cationic polymers can be considered suitable partners for noncovalent interactions with nucleic acids, which resulted in satisfactory in vivo transfection. Cationic polymers provide considerable flexibility in terms of structure modifications and development. Some sequence-defined polymers are advantageous for demonstrating fine structure–activity relationships. Hence, cationic polymers have attracted substantial interest as non-viral delivery systems in the area of nucleic acid delivery. In the last few years, many cationic polymers have been developed, examined, and used as efficient delivery systems for nucleic acids [106]. However, applications of cationic polymers as nanodelivery systems for mRNA have not been thoroughly explored compared to pDNA and siRNA; they have the potential to compete with many well-studied lipid-based delivery systems [107].

#### 2.2.1. Polyplex-Based Nanodelivery Systems

Polyethylenimine (PEI) and its derivatives are some of the best-established polymers as delivery systems for nucleic acids [28,108]. They are water soluble with high contents of protonable amino groups. At neutral extracellular pH, PEI is partly protonated and still binds nucleic acids by electrostatic interactions. When it enters the cells via endocytosis, it resides inside endosomal vesicles and increases its protonation and charge density within acidifying endosomes, leading to osmotic swelling and the subsequent disruption of endosomes and release of the endosomal contents into the cytosol. This is known as the proton sponge effect, which allows nucleic acid escape from the endosomes to the cytosol [109]. Thus, this effect favors the use of PEI in nucleic acid delivery. However, its wide therapeutic use has been hampered by its cytotoxicity due to its high molecular weight (>25 kDa) and highly branched derivatives. This toxicity has been attributed to the adsorption of negatively charged serum proteins, such as albumin, onto the polyplex surface, which causes the polyplexes to aggregate, increasing their effective size [110]. Many studies have been conducted to overcome these challenges. It has been verified that fine-tuning the PEI properties (e.g., molecular weight and ratio) can surmount these issues [51]. Demoulins et al. developed SAM mRNA encoding influenza virus hemagglutinin and nucleocapsid-encapsulated nanoparticles using linear and histidylated PEI (lPEI and his-PEI, respectively) via electrostatic attraction. Flow cytometry and confocal microscopy studies revealed that polyplex promotes SAM RNA’s interaction with dendritic cells and facilitates its translocation to the cytosol. However, his-PEI-polyplexes favor delivery to monocyte-derived dendritic cells over primary porcine blood dendritic cells, whereas lPEI–polyplexes were more consistent for all cells. Analysis of PEI formulation delivery to dendritic cells revealed the translation of encoded influenza virus antigen over a 72h period in vitro. However, his-PEI-polyplexes were less efficient than lPEI–polyplexes at promoting SAM RNA translation. In vivo delivery of PEI formulations enhances SAM RNA translation, as demonstrated by the induced humoral responses against SAM RNA-encoded influenza virus antigens detected in all vaccinates. These humoral responses are augmented in the presence of an adjuvant. PEI formulation delivery promotes strong cellular responses. Hemagglutinin and nucleocapsid-specific memory T-cell activation was observed with and without adjuvant application. Importantly, the PEI formulations enhanced the activation of both humoral and cellular immune responses, while adjuvanted vaccines favored antibody over T lymphocyte stimulation. Polyplex vaccination activated significant levels of systemic cytokines, including IFN-γ (Th1), IL-13 (Th2), IL-6, TNF-α, and IL-17 (Th17). Thus, PEI formulations lead to a balanced Th1/Th2/Th17 response [50].

Given the observed differences between the lPEI and his-PEI polyplexes used in the previous study, the same group optimized the polyplex formulations of lPEI [51]. They modified the PEI molecular weight, the SAM RNA:PEI weight:weight ratio, and included cell-penetrating peptides known for promoting RNA delivery into the cytoplasm [111]. Analysis of this modification revealed how adjusting SAM RNA functionality is crucial, specifically in terms of which antigen-encoded SAM RNAs are made available for translation and the influence on the gene of interest. Interestingly, [SAM RNA/PEI-4k (1:3)] was found to provide the best results for SAM RNA delivery both in vitro and in vivo [51].

High-molecular-weight PEIs may be appropriate for the delivery of SAM RNAs due to their size (12–14 kb) and the complexity of SAM RNA. However, the PEIs high in molecular weight (>25 kDa) are associated with higher cytotoxicity [110]. Delivery systems with large PEI-based polyplexes are usually too stable to liberate mRNA in the cytoplasm [112]. Moreover, the low-molecular-weight PEIs showed poor transfection activity. To mitigate some of these issues, two studies developed a cyclodextrin–PEI polyplex formulations for nasal delivery of mRNA [53,54]. Cyclodextrin complexed with PEI lowered the charge density of the polyamine backbone. Thus, the cytotoxicity of PEI decreased while the protonatable groups were reserved, leading to improved transfection [113]. This PEI alteration enhances mRNA polyplexes to safely overcome epithelial barriers and reach the nasal-associated lymphoid tissue [52], while retaining the good mucosal adjuvanticity of PEI [53].

Li et al. formulated a nasal delivery system composed of β-cyclodextrin which provides the high mucosal affinity and low-molecular-weight PEI 2 k (CP 2k) which provides the good adjuvant property. CP 2k or PEI 25k and mRNA encoding the HIV-1 envelope gp120 subunit were complexed by electrostatic interaction. The average particle size of CP 2k/mRNA was 117.3 ± 3.44 nm at N/P 16 (the molar ratio of nitrogen in PEI portion of CP 2k/phosphate in RNA) with a ζ-potential of 26.4 ± 2.8 mV, and they presented spherical shape. This study compared the ability of naked mRNA, CP 2k, or PEI 25k to deliver mRNA encoding the HIV-1 envelope gp120 subunit and to evoke specific immune responses to gp120. The CP 2k formulation showed longer nasal residence time, which further increased uptake by nasal-associated lymphoid tissue and by nasal epithelial cells. CP 2k enhanced paracellular mRNA delivery and reduced the absorption of toxins present in the nasal cavity via reversible opening of tight junctions in the nasal epithelium. However, PEI 25k irreversibly modified tight junction integrity and permitted bioabsorption of toxins. Intranasal vaccination with CP 2k/mRNA evoked significantly more antibody production than either PEI 25k/mRNA or naked mRNA. CP 2k/mRNA induced significantly higher levels of CD8+ and CD4+ T-cell responses than PEI 25k/mRNA. CP 2k/mRNA led to higher levels of Th1 including IFN-γ and IL-2 and Th2 cytokines including IL-4 and IL-10 than either PEI 25k/mRNA or naked mRNA. Also, CP 2 k/mRNA induced high level of Th17 cytokine (IL-17), indicating that CP 2 k/mRNA evoked strong mucosal and systemic immune responses in a balanced Th1/Th2/Th17 profile. More important, the condensation of mRNA into CP2k polyplexes lower the ability of mRNA to generate an innate immune response. This was demonstrated by the lower generation of type I interferon by CP2k/mRNA polyplexes compared to naked mRNA. However, CP2k/mRNA polyplexes generated moderately higher levels of type I interferon than unimmunized mice. Thus, CP2k/mRNA polyplexes may provide a balance between the antigen-specific immune response and innate immunity due to their ability to evoke a low innate immune response [53,54].

Although most studies of the mRNA polyplex formulations have focused on PEI as a cationic polymer, Coolen et al. recently developed a novel carrier to deliver mRNA vaccines [54]. They used poly(lactic acid) nanoparticles (PLA-NPs) and the cationic cell-penetrating peptides (CPPs) as an intermediate. PLA is a biocompatible, biodegradable polymer that has been approved by the Food and Drug Administration [114] and presents an excellent safety profile [115]. PLA-NPs have demonstrated superiority in the vaccinology field. They provide an adjustable nanodelivery system due to their ability to adsorb and/or encapsulate different antigens and immunostimulant molecules [116,117,118,119]. After parenteral administration, PLA-NPs induce immune responses against different antigens in vivo [116,118]. PLA-NPs are efficiently taken up by dendritic cells, as demonstrated by in vitro and in vivo studies [116,120,121]. PLA-NPs could improve mRNA uptake by dendritic cells [122]. However, a major obstacle facing the efficient delivery of antigen-coding mRNAs into intracellular target sites using PLA-NPs is that both the PLA-NP surface and mRNA are negatively charged. Accordingly, it is essential to reverse the net surface charge of the NP or to design an mRNA intermediate complex with positive charges to adsorb mRNAs onto the surfaces of PLA-NPs. Therefore, one promising strategy is the application of positively charged material as an mRNA intermediate complex.

One study showed that low molecular weight and positively charged polymers form complexes with mRNAs [112]. For the endosomal escape of these complexes and subsequent efficient mRNA translation in the cytosol, a membrane-active peptide must be used. Recently, many reports have suggested that positively charged CPPs might be considered promising delivery systems for mRNA [123]. They offer low positive charge densities and the ability to induce membrane disruption for endosomal escape, which is essential to allow cytosol delivery of mRNA and translation [124]. PLA-NP mRNA-based vaccine formulations were fabricated through the development of an intermediate complex between mRNAs and amphipathic cationic CPPs (RALA, LAH4, or LAH4-L1), followed by adsorption of the intermediate complex onto PLA-NPs. The polyplexes are efficiently adsorbed onto PLA-NPs, as demonstrated by the zeta potential measurements (change from −50 to ≈30 mV). The hydrodynamic diameter changed from ≈188 to ≈240 nm. The uptake of LAH4-L1/mRNA and PLA-NP/LAH4-L1/mRNA nanocomplexes by two epithelial cell lines and dendritic cells was investigated. It was found that LAH4-L1-based formulations failed to transfect epithelial cells. In contrast, they induced strong protein expression in dendritic cells with no toxic effect detected on dendritic cells. These results indicated that the transfection capacity of LAH4-L1-based formulations depends on cell type, suggesting that they are particularly efficient for targeting dendritic cells. The intensity of expression was significantly higher with PLA-NPs/LAH4-L1/mRNA nanocomplexes than LAH4-L1/mRNA polyplexes, indicating that transfection efficiency is enhanced by the presence of PLA-NPs in the formulations. Moreover, LAH4-L1-based formulations are taken up by dendritic cells through phagocytosis and clathrin-dependent endocytosis. These formulations activated both endosome and cytosolic PRRs, leading to activation of the innate immune response. The results indicated the induction of adaptive immune responses in primary human dendritic cells in vitro, including a prevalent Th1 aspect (IFN-γ and IL-12) [54].

#### 2.2.2. Cationic Micelle-Based Nanodelivery Systems

As an alternative to polyplexes, cationic micelles emerged as an efficient gene and peptide delivery system. Cationic micelles are based on an inner core composed of hydrophobic blocks and an outer shell composed of hydrophilic units with mRNA complexed electrostatically in the core [125]. The cationic micelles can self-assemble in aqueous phase and provide competitive advantages, including protection of nucleic acids, promotion of cell uptake, higher gene transfection, and increased safety [126]. The first cationic micelles to deliver mRNA vaccines were developed using stearic acid and branched PEI 2k conjugates (PSA) [55]. The micelles were able to encapsulate HIV-1 gag, successfully encoding mRNA. The characterization of PSA-mRNA micelles revealed particle size and polydispersity indexes of 117.77 ± 3.894 nm and 0.13 ± 0.017, respectively, which suggested the micelles’ formulation was homogeneous. In immunological tests, PSA-mRNA micelles could escape from endosomes and lead to murine bone-marrow-derived dendritic cells’ maturation, as demonstrated by the high level of CD80+. After subcutaneous immunization of PSA-mRNA micelles into mice, the immune responses were notably induced by the formulation compared with naked gag mRNA. The results showed high antigen-specific antibody secretion and pro-inflammatory cytokine production—mainly by IFN-g expressing CD8+ T cells and IL-4 expressing CD4+ T cells. The immune response induced by PSA-mRNA micelles was verified to be a mixed Th1/Th2 response. As fatty acids are safe materials, the safety profile of PSA-mRNA micelles was superior to that of PEI/mRNA complexes, suggesting that these micelles have the capability to provide a safe and efficient vaccine nanodelivery system [55].

#### 2.2.3. Dendrimer-Based Nanodelivery Systems

Dendrimers are typically symmetric around the core. They have highly branched and radial macromolecules similar to dendrites. Dendrimers have many interesting characteristics, for instance, high biocompatibility, predictable biodistribution, and a large number of functional peripheral groups that can interact with biologically active molecules and cell membrane [127]. Dendrimers have a three-dimensional spherical structure that is monodisperse. This feature of dendrimers allows them to pass through cell membranes; thus, they are a better choice as nanodelivery systems than the classical polymers [128]. Due to these unique features, cationic dendrimers have been widely studied for gene delivery [129,130,131]. Among the many types of dendrimers, polyamidoamine (PAMAM) and polypropyleneimine (PPI)-based dendrimers are the most applied delivery systems and have received the most attention [132]. During nanodelivery system synthesis, dendrimers demonstrate a level of control not possible with most linear polymers, resulting in mostly monodisperse, globular macromolecules with a large number of surface functional groups. For the application of functionalized dendrimers as RNA-based vaccine delivery systems, cautious design and examination of their biodistribution, clearance, organ accumulation, and safety profile are essential [133]. The biological features of a dendrimer are mainly controlled by the size and the surface groups of the dendrimer. A direct relationship exists between the generation (size) of the dendrimer and its circulation and degradation time. For a macromolecule, the molecular weight should be above 20 kDa to stay in circulation for a prolonged time, whereas the molecular weight should be below 40 kDa to be secreted through the kidneys to avoid accumulation in the body. These ranges can be tightly governed during the synthesis of dendrimers [134]. The interior structure of the dendrimer is protected to large extent from the external environment by the outer shell and the surface. The dendrimers with peripheral amino groups have a strong tendency to bind to the cell membrane because of their high positive charge density when the generation surpasses G3, leading to high cytotoxicity and induction of destructive cell lysis. Anionic dendrimers have lower affinity to most cell membranes and demonstrate no significant surface-charge-dependent cytotoxicity. The cell membrane interaction with dendrimers with neutral surface charge is influenced by the polar or non-polar end groups of the dendrimers. Polar end groups such as PEG produce a non-toxic and long-circulating particle, whereas non-polar groups such as lipids readily interact with cell membranes and often activate the immune response due to their similarity to bacterial surfaces [135]. The cytotoxicity of toxic cationic dendrimers can be reduced significantly by additives. The G6-PAMAM dendrimer partially modified with the fluorophore Oregon Green and fetal calf serum was less toxic than the unmodified dendrimer. Preparations of dendrimers with ovalbumin demonstrated lower toxicity compared to the dendrimer alone [133]. These results can be explained by the potent shielding effect of the many cationic groups on the surfaces of the dendrimers. Surprisingly, few studies have been conducted on the use of cationic dendrimers as RNA-based vaccine delivery systems, although they have been studied extensively for gene delivery due to their direct electrostatic interaction ability with negatively charged DNA [136]. Khan et al. substituted free amines on multi-generational PAMAM and PPI dendrimers with alkyl chains for siRNA delivery [137]. In a subsequent study, Chahal et al. used the alkyl-chain-modified poly(amido amine) dendrimers to deliver a SAM mRNA vaccine [56,57]. A single dose intramuscularly delivered in mice induced potent CD8+ T-cell and antibody responses and protected mice against different lethal pathogen challenges, including *Toxoplasma gondii*, H1N1 influenza, and Ebola virus. The authors suggested that this modified dendrimer with high charge density could offer maximum protection from nucleases and permit functional release in the cytoplasm compared to lipid vectors [56]. The same group recently examined a similarly designed vaccine against the Zika virus. They found that one dose of intramuscular immunization in mice evoked moderate immune responses followed by a booster vaccination that produced strong and protective immunity [57].

#### 2.2.4. Nanogel-Based Nanodelivery Systems

Polymer nanogels are swollen three-dimensional nano-sized networks constituted of cross-linked hydrophilic or amphiphilic polymer chains. Polymer nanogels are composed of different types of natural polymers, synthetic polymers, or combinations thereof. Their properties, such as charge, size, amphiphilicity, porosity, mechanical strength, and degradability, can be adjusted by changing their chemical composition [133]. Polymer nanogels were originally developed as drug carriers. They can easily absorb biomolecules via noncovalent interactions, for instance, salt bonds, hydrogen bonds, or hydrophobic interactions [138]. One of the main advantages of nanogels is their rapid response to changes in the surrounding environment. These responses can be adjusted by choosing the appropriate polymers and cross-linking agents used for formation of the nanogels. Moreover, polymer nanogels can easily incorporate 30% or more of their weight of oppositely charged biomacromolecules such as DNA and RNA, which is unusually high and exceeds the capacities of liposomes and polymeric micelles [3,81]. As a result of drug loading, the nanogels collapse, forming stable nanoparticles in which biomacromolecules become entrapped, making them good candidates as adjuvant carriers for RNA-based vaccines development [133]. McCullough et al. provided the first description of SAM RNA delivery to dendritic cells by chitosan-based nanogel, which provided both RNase protection and delivery. The delivery system was composed of SAM RNA-loaded chitosan TPP tripolyphosphate nanogel that incorporated chitosan cores into sodium alginate nanogel (NGA). Lipofectamine 2000 incorporated together with chitosan during NGA preparation (NGA-Lipo) was used as a positive control. According to the RNase resistance assay results, chitosan protected labeled RNA probes against RNase. Thus, NGA, NGA-Lipo, and a chitosan core overcome the inability of naked SAM RNA to survive in biological environments and promote intracellular delivery to dendritic cells. In addition, the results showed that the translocation and translation of SAM RNA are dependent on SAM RNA concentration and occur in a kinetic manner. In vivo studies showed an effective translocation of SAM RNA by the chitosan core as well as NGA. NGA-Lipo delivery in vivo was not effective, as observed in in vitro studies [58].

### 2.3. Hybrid-Based Nanodelivery Systems

The nanodelivery systems for mRNA vaccines may consist of many chemical compounds, including lipids, polymers, and peptides in one structure for more potent transfection. These nanodelivery systems can be classified as hybrid nanodelivery systems. These hybrid nanodelivery systems normally integrate the potential advantages of their constituents and offer more flexibility in comparison with non-hybrid nanodelivery systems [27,32].

#### 2.3.1. Cationic Nanoemulsion-Based Nanodelivery Systems

Brito et al. from Novartis Institutes developed a hybrid cationic nanoemulsion for delivering SAM mRNA-based vaccinations. This nanoemulsion is based on the company’s proprietary adjuvant MF59, which is formulated by combining and heating oil-phase constituents (squalene and sorbitan trioleate) to 37 °C, followed by mixing with aqueous phase constituents (Tween-80 in citrate buffer at pH 6.5). The nanoemulsion adjuvant MF59 is well tolerated in children, adults, and the elderly, and has demonstrated a clinically safe profile [139]. It became the second adjuvant available for commercial use after aluminum. The researchers used MF59 as a vaccine base and they added DOTAP to the oil phase to electrostatically bind the SAM mRNA. The final emulsion had a small size, below 100 nm, as measured by dynamic light scattering. In vivo studies demonstrated that delivery of the SAM mRNA vaccine induced potent immune responses in different animal models (mice, rats, rabbits, and non-human primates) against respiratory syncytial virus, human immunodeficiency virus, and human cytomegalovirus. These immune responses evoked using a cationic emulsion were comparable to a viral delivery system. After intramuscular injection, this formulation improved the local immune response by recruiting immune cells, similarly to MF59 adjuvant subunit vaccines [59]. One advantage of cationic nanoemulsions is that their constituents have already been applied in previous clinical studies [140]. Further studies demonstrated the immunogenicity of this cationic nanoemulsion against different viruses in different animal models, including influenza virus hemagglutinin (HA) in ferrets and mice and HIV-1 in rhesus macaques [60,61]. Using the same preparation, a recent study demonstrated the immunogenicity and moderate protective efficacy of the cationic nanoemulsion against bacterial pathogens, namely *Streptococcus* (groups A and B) spp. in mice [62].

#### 2.3.2. Lipopolyplex-Based Nanodelivery Systems

The lipopolyplex is a ternary complex structure composed of a lipid shell surrounding a preformed nucleic-acid–polycation complex core. Although lipopolyplexes were originally used for siRNA [141,142,143,144] or DNA [145] transfection [146], their utility as nanodelivery systems for mRNA delivery has only recently been investigated. Lipopolyplexes combine the advantages of lipoplexes and polyplexes into one entity. This nanodelivery system offers an effective alternative by taking advantage of polyplex properties, such as small particle size, homogeneity, endosomal escape, and high transfection activity. High stability, low cytotoxicity, and acceptable cellular uptake, which are usually associated with lipoplexes, are gained [147], and they can perfectly protect mRNA from degradation.

The earliest works on mRNA-based vaccine delivery by lipopolyplexes were reported by Hoerr et al. They complexed mRNA encoding β-galactosidase with polycationic peptide protamine, forming a stable polycation–mRNA complex, and encapsulated the complexes with liposome. In vitro, this lipopolyplex formulation protected the encapsulated mRNA for a longer period of time and showed in vivo protein expression and provoked an immune response [148]. Recently, Mai et al. designed a similar system composed of a protamine–mRNA complex encapsulated into DOTAP/Chol/DSPE-PEG cationic liposomes. The mRNA–protamine–cationic liposome system effectively promoted vaccine uptake by dendritic cells. This formulation also exhibited stronger capacities to stimulate dendritic cell maturation and to promote cytokine secretion, leading to a potent anti-tumor immune response. In vivo studies showed that the intranasal delivery of the mRNA–protamine–cationic liposome system is capable of eliciting a strong cellular immune response and slowing tumor growth in an aggressive Lewis lung cancer model [63].

Many pH-sensitive polymers that demonstrate an endosome-disrupting function due to the decrease in pH upon endocytosis have been examined for mRNA hybrid nanodelivery systems. Mockey et al. developed histidylated lipopolyplexes consisting of histidylated cationic liposomes and PEGylated histidine-rich polymers for mRNA vaccine delivery against melanoma. mRNA encoding the MART1 antigen was complexed by PEGylated histidine-rich polymers to form polyplexes and entrapped in liposomes. The cationic charges in polyplexes were reduced by the presence of the PEG molecule on the cationic polymer. This resulted in PEG-polyplex entrapment by the cationic liposomes due to reduction of the repulsive force between polyplexes and cationic liposomes. Some advantages of this delivery system are the protection of mRNA against degradation and the ability to enhance endosome escape of mRNA inside APCs in a pH-responsive manner. Intravenous injection of this mRNA encoding MART1 histidylated lipopolyplexes led to specific and significant protection against B16F10 melanoma tumor progression and reduced lung metastasis formation in mice, in contrast to corresponding lipoplexes or polyplexes [64].

Targeting dendritic cells through the overexpressed surface receptors/ligands has also been investigated in mRNA-based vaccines. For instance, the mannose receptors have been targeted by mannosylated-based lipopolyplexes. The first type of mannosylated lipopolyplex was constructed with a cationic polymer to condense mRNA and the mannosylated lipid. The cationic polymer used was PEGylated histidylated polylysine (PEG-HpK). The lipid shell composed of lipophosphoramidate liposomes was formed with histamine lipophosphoramidate (protonable lipid), N-methyl imidazolium lipophosphoramidate (cationic lipid), and a mannosylated lipid to enhance capture and uptake by dendritic cells [65,94]. The results demonstrated that the delivery of mRNA to dendritic cells was improved by the mannose residue on the mannosylated lipopolyplexes through the interaction with the mannose receptor. In vivo studies demonstrated that after intravenous administration of lipopolyplexes, 3% of splenic dendritic cells were expressing the antigen. This value was further increased to 13% using mannosylated lipopolyplexes, with no cytotoxicity noticed in vivo. A greater inhibition of B16F10 melanoma growth was obtained, suggesting that mannosylated lipopolyplexes provide an efficient mRNA delivery system to dendritic cells [65].

The second type of mannosylated lipopolyplexes add a glycolipid including a tri-antenna of a-D-mannopyranoside to replace monovalent mannose to improve capture and uptake by dendritic cells [149]. Cationic and mannosylated lipopolyplexes demonstrated potent anti-tumor effects in several tumor models when used for therapeutic vaccines [66,67]. Recently, Perche et al. developed neutral mannosylated lipopolyplexes with mRNA or SAM RNA. These formulations were stable in media containing serum and they efficiently transfected dendritic cells in cellulo. The results showed that intravenous injection of neutral mannosylated lipopolyplex–mRNA complexes led to reporter protein expression in mice. Intramuscular injection of neutral mannosylated lipopolyplex–SAM RNA complexes encoding an influenza antigen led to sustained gene expression in vivo and induced functional antigen-specific T cells [68].

Another pH-sensitive polymer, poly(β-amino ester) (PbAE), has been investigated for a range of gene delivery applications. PbAE is an ionizable and biodegradable polymer that can be easily synthesized [150,151]. Su et al. formulated a PbAE core embedded into a phospholipid bilayer shell for in vivo delivery of mRNA. The PbAE constituent was selected to enhance endosome disruption. The DOTAP-containing lipid surface layer was selected to reduce the toxicity of the polycation core and to efficiently adsorb mRNA via electrostatic interactions onto the surfaces of these cationic nanoparticles. In vitro studies showed that this hybrid nanoparticle is efficiently taken up by dendritic cells with cytosol location and low cytotoxicity. The intranasal administration of these hybrid nanoparticles led to the expression of the reporter protein as soon as 6h after administration, whereas naked mRNA displayed no signal [69].

In another report, mRNA was complexed with PbAE to form a complex core, which was then entrapped into a bilayer lipid shell to form a lipopolyplex. This hybrid mRNA vaccine delivery system demonstrated intrinsic adjuvant activity by strongly stimulating INF-b and IL-12 expression in dendritic cells through Toll-like receptor 7/8 signaling. It also improved the antigen-presenting ability of dendritic cells. Subcutaneous administration of this lipopolyplex formulation led to tumors being shrunk by over 90% in mice with lung metastatic B16-OVA tumors [70]. However, the PbAE-complexed mRNA system just slightly evoked IFN-γ secretion in vivo for vaccine applications [152]. Recently, Guevara et al. incorporated the immune adjuvant α-galactosylceramide (α-GalCer) into a lipopolyplex delivery system for the in vivo delivery of mRNA into APCs [71]. α-GalCer, also known as KRN7000, is one of the optimal new classes of vaccine adjuvants due to its ability to link between innate and adaptive immunity. α-GalCer is an invariant natural killer T (iNKT) cell antigen presented on the CD1d of APCs. Previous studies have indicated that the stimulation of iNKT cells evokes the cytotoxic T lymphocytes-generated elimination of tumor cells or different infections [153]. The developed lipopolyplex formulation was composed of a PbAE/mRNA polyplex core surrounded by a lipid shell. PbAE has the ability to condense the mRNA into a polyplex nanoparticle via an electrostatic interaction. The lipid shell consists of a multivalent cationic lipid (MLV5), 1,2-dioleoyl-sn-glycero-3-phosphoethanolamine (DOPE), DSPE-PEG, and α-GalCer to enhance mRNA delivery into dendritic cells. The lipid shell improves the immune response due to the adjuvant activity mediated by α-GalCer. The α-GalCer-/mRNA-loaded lipopolyplex targeted dendritic cells after intravenous administration without the need for its functionalization with cell-specific antibodies or ligands. In vitro and in vivo studies demonstrated that the α-GalCer-/mRNA-loaded lipopolyplex efficiently led to high expression of the enhanced green fluorescence protein in dendritic cells, exhibiting an intrinsic selectivity for dendritic cells. The TRP2-mRNA/α-GalCer-loaded lipopolyplex induced a significant therapeutic effect in B16-F10 melanoma-bearing mice [71]. Unfortunately, the lipid-based delivery systems enable easy integration of lipid-like adjuvants, but not other adjuvants, such as Toll-like receptor 7 (TLR7) ligands. TLR7 ligands are one class of promising potent adjuvants for anticancer immunotherapy. Alternatively, a poly(lactic-co-glycolic acid) (PLGA) core/lipid-shell nanoparticle was developed as a delivery system. This delivery system allows for efficient adjuvant loading of hydrophobic TLR7 adjuvants such as gardiquimod into the PLGA core, while the lipid shell allows the complexation of mRNA. This hybrid nanovaccine demonstrated effective antigen expression and dendritic cell activation in vitro. In vivo studies demonstrated spleen enhancement of mRNA and a strong immune response after intravenous administration of this formulation. The co-delivery of the antigen and adjuvant by the hybrid nanovaccine showed anti-tumor activity in both therapeutic and protective models employing B16-OVA [72].

## 3. Conclusions and Future Perspectives

Based on preclinical and clinical studies, mRNA-based vaccines using nanodelivery systems show promise as tools for the evolution of novel therapeutic and prophylactic vaccines against infectious diseases and cancer. Importantly, mRNA-based vaccines are good candidates for responding to the COVID-19 pandemic. The design and manufacturing of mRNA-based vaccines on the clinical scale are possible within weeks when the viral antigen sequence becomes available. However, many obstacles are facing the development of mRNA-based vaccines using nanodelivery systems, such as the high molecular weight of mRNA, negatively charged mRNA, intrinsic instability, and the high susceptibility to degradation by ribonuclease. Therefore, nanodelivery systems are crucial for the successful in vivo delivery of mRNA to the site of action. Currently, lipid-based nanodelivery systems are mostly used for developing mRNA-based vaccines. Polymers and lipid–polymer hybrid nanodelivery systems show considerable promise in terms of stability, high transfection efficiency, safety profile, and cost. Advances in mRNA nanodelivery systems using different materials can keep pace with the urgent need for prophylactic vaccines during pandemics. Although many developed mRNA vaccine formulations are stored frozen (−70 °C), efforts to develop thermostable formulations more suitable for vaccine distribution has been gaining interest. Published studies imply that stable refrigerated or room temperature formulations can be developed. One study reported that the activity of freeze-dried mRNA with trehalose stored at 5–25 °C for 36 months and at 40 °C for 6 months was not compromised [2]. Another lyophilized mRNA vaccine was shown to be stable at 4 °C for at least 10 months [154]. Furthermore, when a protamine-complexed mRNA vaccine was subjected to oscillating temperatures between 4 and 56 °C for 20 cycles and exposure 70 °C it retains its full biological activity [155]. Enhancing thermostability for long-term storage at high temperatures is an important feature to be further studied to enable easier distribution and storage at rural areas and developing countries.

## Figures and Tables

**Table 1:** Examples of clinical trials using nanodelivery systems for mRNA-based vaccines.

Disease	Target Antigen	Route/Nanodelivery System Type	Phase	Status/Results	Reference
Rabies	Rabies virus glycoprotein	IM; ID; protamine complex	I	(Completed) Produced boostable neutralizing antibodies when administered with a needle-free device (spring-powered ID and IM injectors, carbon dioxide gas-powered ID injector); vaccine appeared to be safe, with no serious adverse effects except for a case of temporary moderate Bell’s palsy	[2] NCT02241135
H10N8 and H7N9 Influenza Viruses	Hemagglutinin glycoprotein from the H10N8 influenza strain or the H7N9 influenza strain	IM; ID; lipid nanoparticles	I	(H10N8 vaccine: Completed; H7N9 vaccine: Active, not recruiting). Vaccines produced a strong humoral immune response in healthy adults with no serious adverse effects	[3] NCT03345043
COVID-19	SARS-CoV-2 spike (S) glycoprotein	IM; lipid nanoparticles	I	(Active, not recruiting) Provoked high levels of binding and neutralizing antibodies in younger and older adults and the responses were similar those seen in COVID-19-recovered patients; no serious adverse effects were reported	[3,4] NCT04283461
	SARS-CoV-2 spike (S) glycoprotein	IM; lipid nanoparticles	I/II	(Recruiting) No results posted	NCT04480957
	Two vaccines: BNT162b1, encoding a secreted trimerized SARS-CoV-2 receptor-binding domain; BNT162b2, encoding a prefusion stabilized membrane-anchored SARS-CoV-2 full-length spike	IM; lipid nanoparticles	I/II	(Recruiting) Both vaccines stimulated neutralizing antibodies in younger and older adults that are similar or higher than COVID-19-recovered patients; BNT162b2 was associated with less systemic reactions, especially in older participants; no serious adverse effects were reported	[5,6] NCT04368728
Prostate Cancer	Prostate-specific membrane antigen, prostate stem cell antigen, and six-transmembrane epithelial antigen of the prostate 1	ID; protamine complex	I/IIa	(Terminated) Induction of CD4+ and CD8+ T-cell responses; probably vaccine-related urinary retention occurred in 3 patients	[7] NCT01817738
Non-small Cell Lung Cancer	Non-small cell lung cancer antigens: New York esophageal squamous cell carcinoma (NY-ESO-1), melanoma antigen family (MAGE) C1 and C2, baculoviral inhibitor of apoptosis repeat-containing 5, trophoblast glycoprotein, and mucin-1 antigen	ID; protamine complex	Ib	(Terminated) Induction of immune response against the six encoded antigens; no vaccine-related serious adverse effects were reported	[8] NCT01915524
Melanoma	NY-ESO-1, MAGE-A3, tyrosinase and TPTE	Intravenous; lipoplex	I	(Active, not recruiting) Induction of IFN-α and strong antigen-specific T-cell responses	[9] NCT02410733

mRNA: messenger RNA; ID: intradermal; IM: intramuscular; COVID-19: coronavirus disease 2019; SARS-CoV-2: Severe acute respiratory syndrome coronavirus 2; IFN-α: interferons-α.

**Table 2:** Examples of recent nanodelivery systems for mRNA-based vaccines.

Nanodelivery System Type	Nanodelivery System Compositions	RNA Type	Target	Route of Administration	In Vivo Model	Adjuvant	Reference
**Lipid-based Nanodelivery Systems**							
Lipid nanoparticles	Ionizable lipid, phospholipid, cholesterol, PEG lipid	mRNA	Influenza virus, rabies virus	Intramuscular	Non-human primates	None	[40]
	Ionizable lipid, DSPC, cholesterol, PEG lipid	mRNA	Influenza virus	Intramuscular	Rodent and non-human primates	None	[41]
	Ionizable lipid, DSPC, cholesterol, PEG lipid	mRNA	COVID-19	Intramuscular	Mice and non-human primates	None	[42]
	Ionizable lipid, DSPC, cholesterol, PEG lipid	mRNA	Respiratory syncytial virus	Intramuscular	Mice and cotton rats	None	[43]
	Ionizable lipid, DSPC, cholesterol, PEG lipid	mRNA	Zika virus	Intramuscular	Mice	None	[44]
	DC-Chol, DDA, DOTAP, DMTAP, DSTAP, DOBAQ, DMG-PEG2000	SAM RNA	Rabies virus	Intramuscular	Mice	None	[45]
	DOTAP, DOPE, DSPE-mPEG2000), Mannose	mRNA	Influenza virus	Intranasal	Mice	None	[46]
Lipoplexes	Cationic liposomes	SAM RNA	Influenza virus	Subcutaneous	Mice	PEGylated MALP-2	[47]
	DOTAP liposomes, cholesterol-modified cationic peptide DP7	mRNA	Subcutaneous tumors	Subcutaneous	Mice	None	[48]
	InstantFECT (liposome-based transfection reagent)	mRNA	*Staphylococcus aureus* or B16-OVA tumor	Intratumoral, subcutaneous, intramuscular	Mice	None	[49]
**Polymer-based Nanodelivery Systems**							
Polyplexes	Linear or histidylated Polyethylenimine	SAM RNA	Influenza virus	Subcutaneous	Mice	Pam3Cys-SK4 (P3C) or BPPcysMPEG (BPP)	[50]
	Polyethylenimine and cell-penetrating peptides	SAM RNA	Influenza virus	Intrapulmonary intradermal	Mice pigs	c-di-AMP	[51]
	Polyethylenimine and cyclodextrin	mRNA	HIV-1	Intranasal	Mice	None	[52]
	Polyethylenimine and cyclodextrin	mRNA	Ovalbumin	Intranasal	Mice	None	[53]
	Poly(lactic acid) and cell-penetrating peptides	mRNA	HIV-1	N/A	N/A	None	[54]
Cationic micelles	polyethyleneimine stearic acid	mRNA	HIV-1	Subcutaneous	Mice	None	[55]
Modified dendrimer nanoparticle	Modified dendrimer	SAM RNA	Influenza virus, Ebola virus, *Toxoplasma gondii*	Intramuscular	Mice	None	[56]
	Modified dendrimer	SAM RNA	Zika virus	Intramuscular	Mice	None	[57]
Nanogel	Chitosan and sodium alginate	SAM RNA	Influenza virus	Subcutaneous	Mice and rabbits	PEGylated MALP-2	[58]
**Hypride-based Nanodelivery Systems**							
Cationic nanoemulsion	DOTAP and emulsion adjuvant MF59	SAM RNA	Respiratory syncytial virus, human cytomegalovirus and HIV	Intramuscular	Mice, rabbits, Rhesus, macaques	Emulsion adjuvant MF59	[59]
	DOTAP and emulsion adjuvant MF59	SAM RNA	HIV	Intramuscular	Rhesus macaques	Emulsion adjuvant MF59	[60]
	DOTAP and emulsion adjuvant MF59	SAM RNA	Influenza Virus	Intramuscular	Mice ferrets	Emulsion adjuvant MF59	[61]
	DOTAP and emulsion adjuvant MF59	SAM RNA	Group A and Group B Streptococci	Intramuscular	Mice	Emulsion adjuvant MF59	[62]
Lipopolypelexs	*Protamine* DOTAP/Chol/DSPE-PEG	mRNA	Lung cancer	Intranasal	Mice	None	[63]
	PEGylated histidylated polylysine L-histidine-(N,N-di-n-hexadecylamine)ethylamide and cholesterol	mRNA	Melanoma	Intravenous	Mice	None	[64]
	PEGylated histidylated polylysine mannosylated liposomes	mRNA	Melanoma	Intravenous	Mice	None	[65]
	PEGylated histidylated polylysine Tri-mannosylated liposomes	mRNA	Melanoma lymphoma	Intradermal intravenous subcutaneous	Mice		[66]
	PEGylated histidylated polylysine tri-mannosylated and imidazoylated liposomes	mRNA	Melanoma	Intravenous	Mice	None	[67]
	Polyethylenimine tri-mannosylated anionic liposomes	mRNA or SAM RNA	Influenza	Intravenous or Intramuscular	Mice	None	[68]
	Poly(β-amino ester), phospholipid	mRNA		Intranasal	Mice	None	[69]
	Poly(β-amino ester), phospholipid	mRNA	Lung metastatic B16-OVA tumor	Subcutaneous	Mice	None	[70]
	Poly(β-amino ester), phospholipid	mRNA	Melanoma	Intravenous	Mice	α-galactosylceramide	[71]
	Poly(lactic-co-glycolic acid), phospholipid	mRNA	Melanoma	Intravenous	Mice	Toll-like receptor 7	[72]

mRNA: messenger RNA; SAM RNA: self-amplifying RNA; DSPC: distearoylphosphatidylcholine; PEG: polyethylene glycol; COVID-19: coronavirus disease 2019; DC-Chol: 3ß-[N-(N’,N’-dimethylaminoethane)-carbamoyl]cholesterol; DDA: dimethyldioctadecylammonium; DOTAP: 1,2-dioleoyl-3-trimethylammonium-propane; DMTAP: 1,2-dimyristoyl-3-trimethylammonium-propane; DSTAP: 1,2-stearoyl-3-trimethylammonium-propane; DOBAQ: N-(4-carboxybenzyl)-N,N-dimethyl-2,3-bis (oleoyloxy)propan-1-aminium; DMG-PEG2000: 1,2-dimyristoyl-sn-glycero-3-phosphoethanolamine-N-[methoxy(polyethylene glycol)-2000]; DOPE: 1,2-dioleoyl-sn-glycero-3-phosphoethanolamine; DSPE-mPEG2000: 1,2-distearoyl-sn-glycero-3-phosphoethanolamine-N-(methoxy (polyethylene glycol)-2000); OVA: ovalbumin.

## Data Availability

No new data were created or analyzed in this study. Data sharing is not applicable to this article.
